# Integration of in vitro allergy test results and ratio analysis for the diagnosis and treatment of allergic patients (INTEGRA)

**DOI:** 10.1002/clt2.12052

**Published:** 2021-09-16

**Authors:** Mariona Pascal, Carmen Moreno, Ignacio Dávila, Ana I. Tabar, Joan Bartra, Moisés Labrador, Olga Luengo

**Affiliations:** ^1^ Immunology Department Centre de Diagnòstic Biomèdic Hospital Clínic de Barcelona Institut d'Investigacions Biomèdiques August Pi i Sunyer (IDIBAPS) Universitat de Barcelona Barcelona Spain; ^2^ ARADyAL Network Health Institute Carlos III Madrid Spain; ^3^ Allergy Service Hospital Universitario Reina Sofía Maimonides Biomedical Research Institute of Córdoba (IMIBIC) Córdoba Spain; ^4^ Allergy Service Department of Biomedical and Diagnostic Sciences and Institute for Biomedical Research of Salamanca (IBSAL) University Hospital of Salamanca Salamanca Spain; ^5^ Allergy Service Hospital Complex of Navarra Pamplona Spain; ^6^ Navarra Institute for Health Research (IdiSNA) Cooperative Health Research Thematic Networks (RETICs) for Asthma Madrid Spain; ^7^ Allergy Section, Pneumology Department Hospital Clínic de Barcelona Institut d'Investigacions Biomèdiques August Pi i Sunyer (IDIBAPS) Universitat de Barcelona Barcelona Spain; ^8^ Allergy Section Internal Medicine Department Hospital Universitari Vall d´Hebron Institut de Recerca Vall d'Hebron (VHIR) Universitat Autònoma de Barcelona. Barcelona Spain

**Keywords:** allergy, IgE, IgE ratio, molecular diagnosis, recommendations

## Abstract

The introduction of molecular diagnosis into routine clinical practice has substantially improved the diagnosis and management of allergic patients by allowing clinicians to precisely identify the allergenic molecule responsible for immunoglobulin E (IgE)‐mediated allergies. However, it can be challenging to accurately interpret the results of molecular assays, partly due to the limited evidence base. In this context, a panel of experts with extensive experience in interpreting in vitro measures of total and serum specific IgE reviewed the available scientific evidence. After this review, the panel selected a series of representative case studies to demonstrate how determination of specific and total IgE values and the relationship between them (ratio analysis) can add value to the diagnostic process by more precisely defining the patient’s sensitization profile. Finally, the experts developed a series of recommendations on the clinical application of ratio analysis to optimize and complement the classical approach to allergy diagnosis.

## 1 INTRODUCTION

Allergic diseases are the most common chronic conditions in Europe, affecting an estimated 150 million people. By the year 2025, more than 50% of the European population is expected to manifest some type of allergy.^1^ Given the growing incidence of allergic disease and the increasing complexity of determining the main sensitizing allergens,^2^ it is now more important than ever to ensure diagnostic accuracy for the optimal management of allergies. The diagnostic process has two main components: (1) comprehensive evaluation of the patient’s medical history and (2) diagnostic testing, including the sensitization profile determined by in vivo and in vitro techniques.^3^

The most important in vitro diagnostic tests are total serum immunoglobulin E (tIgE) and serum specific IgE (sIgE) against the whole extract (we‐sIgE) or allergen molecules (c‐sIgE). IgE antibody levels are compared to the International Reference Preparation for Human IgE, a standard curve developed by the World Health Organization.^4^ In addition to measuring and evaluating the clinical significance of each variable (sIgE and tIgE) separately, analysing the relationship between them (ratio analysis) can also provide valuable information to improve their clinical interpretation.^5^ Similarly, allergen‐sIgE can be compared to the we‐sIgE or c‐sIgE.^6^

For these purposes, two ratios have been proposed:

- Ratio 1 (we‐sIgE/tIgE): the analysis of the whole extract serum‐specific IgE/total IgE ratio^5^ allows to determine the sensitization attributable to the whole extract, expressed as a percentage or ratio. This ratio is particularly valuable in two clinical scenarios: (1) atopic patients with extremely low tIgE values (<20 kU/L) and (2) patients with extremely high tIgE values.^5^
- Ratio 2 (c‐sIgE/we‐sIgE): It allows to determine the extent to which a given allergenic component is responsible for sensitization to a whole extract. Ratio 2 is useful in two clinical scenarios: (1) sIgE positive for the whole extract but negative for all major components; this finding suggests that the patient may be sensitized to an untested or unknown component; (2) sIgE negative for the whole extract but positive for a specific component; this finding is commonly observed when a given component (e.g., lipophilic components such as oleosins, defensins, and other) is underrepresented in the whole extract.^3^ In addition, because quantitative differences may be present, it may be helpful to calculate and analyse these ratios to help refine the diagnosis.

For both ratios, the same technique must be used to determine the different IgE values.

### 1.1 Classical approach to allergy diagnosis

Allergy diagnosis begins with a detailed medical history and physical examination, the findings of which may strengthen the diagnostic suspicion. In addition to the usual tests (lung function tests, radiological studies, and other) and specific complementary tests for allergy, skin prick tests (SPT) and – less frequently – intradermal tests (e.g., for Hymenoptera venom) are performed. The test results can help with the diagnostic orientation (aetiology) and thus guide selecting the most appropriate in vitro sIgE tests. In some cases, such as allergies to certain drugs or Hymenoptera venom, other tests – such as the basophil activation test (BAT) or measurement of serum tryptase levels – can provide valuable diagnostic information. The final diagnostic step is controlled in vivo challenge testing, the reference standard in the diagnosis of allergy.^3^, ^7^

Although the diagnostic procedure described above applies to most cases, the diagnostic process should be adapted to each case. For example, in cases of anaphylaxis, it is essential to determine the underlying allergy and rule out mast cell activation syndrome (MCAS) by determining serum tryptase levels at baseline and during the acute phase. Other complementary tests may also be necessary. Serum‐sIgE tests are designed to detect and measure the concentration of IgE to a specific allergen.^6^ Positive results in sIgE tests indicate the culprit allergens and are helpful to recommend avoidance measures (which often alleviate symptoms)^8^ and for diagnostic purposes. These tests can also provide valuable data, in some instances, to evaluate the effects of treatment.

### 1.2 Challenges in interpreting the results of in vitro allergy tests

It is essential to collect as much data as possible to establish an accurate diagnosis. However, the individual test results should be considered in the overall context of the entire battery of tests,^9^ although, this can be a highly complex and challenging process. For example, experiments involving the allergenic protein *Der p 2* have demonstrated that several different factors (sIgE levels, affinity, clonality, and the sIgE/tIgE ratio) influence the effector cell response.^10^ Thus, determination of sIgE alone (vs. the we‐sIgE or c‐sIgE) might not be sufficient to make an accurate diagnosis. In some cases, it would be beneficial to evaluate other factors (affinity, clonality, and others), but due to cost and time restraints, this is generally not feasible in routine clinical practice. For example, in many cases, tIgE is not measured due to the overlap between atopic and non‐atopic patients, test‐related expense,^11^ and the limited value of tIgE as a predictor of sensitization to the most common aeroallergens.^12^ However, for diagnostic purposes, the relationship between tIgE and sIgE is important since the sIgE level's clinical relevance depends on its fractional relation to tIgE when determining receptor occupancy rate of effector cells.^13^ For example, allergen‐sIgE levels representing only 1% of tIgE may be sufficient to trigger half of the maximal degranulation response of effector cells,^5^, ^10^ which could be sufficient to explain the patient’s clinical symptoms.

Another challenge is the difficulty of differentiating between cross‐reactivity and genuine sensitization. In this case, molecular diagnosis, together with the comparative assessment of IgE values, can help determine if sensitization is due to cross‐reactivity and its clinical significance.^5^ In some cases, it is important to identify the specific allergen(s) and allergenic component(s) to which the patient is sensitized in order to select the appropriate allergen‐specific immunotherapy (AIT) and to predict possible side effects.^14^

The present expert consensus statement aimed to compile the available evidence on two ratios – we‐sIgE/tIgE (ratio 1) and c‐sIgE/we‐sIgE (ratio 2) – to elaborate a series of recommendations about the application of these ratios and to discuss their utility in daily clinical practice. These two relations can provide valuable information to optimize the diagnosis of allergic patients, complementing standard diagnostic procedures. In addition, through a series of case studies involving common clinical scenarios, we illustrate how ratio analysis can improve diagnostic accuracy.

## 2 MATERIAL AND METHODS

In June 2019, an expert panel of clinicians ‐the authors of this manuscript‐ with extensive experience in the management and diagnosis of allergy met to define the scope of this work (see Supporting information). Next, these professionals performed a non‐exhaustive, systematic bibliographic search in PubMed to identify relevant articles published in English and Spanish in the last 10 years. The following search terms were used: (1) specific IgE; (2) total IgE; (3) whole extract; (4) IgE ratio; (5) allergy; (6) in vitro IgE. To identify other relevant studies, the authors reviewed the references of the articles identified through the PubMed search and finally contributed with articles of interest not identified through the aforementioned search process.

The authors then selected a series of representative case studies to illustrate how ratio analysis can enhance the diagnostic process. After analysis and synthesis of the evidence, the experts drafted and validated a series of recommendations, which were externally validated by an external group of specialists in allergy management and molecular diagnosis (*n* = 28) through a two‐round Delphi technique. The external group of specialists was asked to indicate their level of agreement with the proposed recommendations on a four‐point Likert scale ranging from 1 (strongly disagree) to 4 (strongly agree). Recommendations supported by ≥ 70% of respondents (score of 3 and 4) were approved. One recommendation failed to reach the 70% threshold in the first round and was reviewed by the expert panel and modified as necessary based on the suggestions made by the external group specialists. The modified recommendation was re‐evaluated in a second round following the same criteria and process as described above. The approval rate in the first round was 85% (6/7) and 100% (1/1) in the second round.

## 3 RESPIRATORY ALLERGY (ASTHMA AND RHINOCONJUNCTIVITIS)

Respiratory allergy is a condition that, in most cases, does not pose a risk to the patient's life, although it often negatively impacts the quality of life (QoL) and, potentially, school and work productivity.^15^ The impact of allergy on QoL is mainly attributable to chronicity and lack of control (>50% of cases).^16^ In most patients (80%), asthma is associated with comorbid rhinitis or rhinosinusitis and, in many cases, conjunctivitis. The comorbidity rate increases as a function of disease severity.^17^ Although measures such as allergen avoidance (not always feasible) and quitting smoking can relieve symptoms, the main treatment of respiratory allergies such as asthma is pharmacological. In many cases, allergen hypersensitivity can be reduced by AIT.^15^, ^18^, ^19^

In cases of severe respiratory disease, the phenotype should be determined during the diagnostic process. This is important because the phenotype can condition the treatment approach (e.g., biologics) and the prognostic implications.^20^ In 60%–90% of cases, the disease is associated with an allergic trigger.^21^ Consequently, it is essential to determine sensitization to inhaled allergens and to assess the clinical relevance of test results.^22^

### 3.1 Respiratory allergy (asthma and rhinoconjunctivitis) to house dust mites and fungal spores

In some cases, the primary sensitizing allergen molecule may not be included in the AIT for dust mites; consequently, it is essential to determine the main sensitizing component to select the most appropriate AIT composition.^14^, ^23^, ^24^ In a study involving patients receiving AIT containing *Alternaria alternata* or dust mite extracts, di Lorenzo et al. showed that a ratio 1 (we‐sIgE/tIgE) value > 16.2 in monosensitized patients was associated with a better response to AIT. This finding demonstrates the value of assessing this ratio compared to sIgE or tIgE values alone.^25^

In clinical practice, many patients test positive for various house dust mite species.^26^ Dust mites can cross‐react with other allergenic sources, such as crustaceans, due to tropomyosin and other common allergens.^27^ The clinical relevance of this cross‐reactivity can be determined by assessing ratio 2 (c‐sIgE/we‐sIgE), which considers c‐sIgE tropomyosin values relative to the we‐sIgE for a given mite species.^28^ Another good example that highlights the utility of ratio 2 is the prescription of AIT to treat allergy to *Alternaria alternata*. In these cases, it is essential to demonstrate sensitization to *Alt a 1* and ensure the relevance of that sensitization by evaluating the allergen‐sIgE (*Alt a 1*) to we‐sIgE (*Alternaria alternata*) ratio.^29^

Other ratios have also proven valuable as predictors of response to AIT. Li et al. found that the c‐sIgE/tIgE ratio for *Der p 2* and *Der f 2* was significantly associated with a favourable clinical response to AIT. Several other factors were significant predictors, including tIgE, tobacco smoke exposure, and family history of atopy.^30^

A case study is described in Table 1 to illustrate these two clinical scenarios.

**TABLE 1:** Case study of a patient with respiratory allergy (asthma and rhinoconjunctivitis) to house dust mites

Case study	
25‐year‐old patient with persistent moderate rhinoconjunctivitis and mild intermittent bronchial asthma with exacerbations in humid regions, with spring and autumn seasonality	
Skin prick testing	
Positive for house dust mite allergy (*Dermatophagoides pteronyssinus* and *D. farinae*).	
Serological test results	
• tIgE: 152 kU/L	
• we‐sIgE *D. pteronyssinus*: 130 kU_A_/L	
• sIgE Der p 1: 1.8 kU_A_/L	
• sIgE Der p 2: 3.2 kU_A_/L	
• sIgE Der p 23: 68 kU_A_/L	
Ratio analysis	
Ratio 1 we‐sIgE/tIgE	This ratio is close to one, which means that the patient's sensitization is mainly due to *D. pteronyssinus*.
Ratio 2 c‐sIgE/we‐sIgE	If Der p 1 and Der p 2 were considered alone, the positive values could suggest that sensitization to mites is attributable to these components. However, when we calculate ratio 2, the low percentage of them versus the whole extract – 1.38% and 2.46%, respectively – suggest that the sensitization must be attributable to other molecular components. A subsequent study demonstrated that the patient presents a clear sensitization to Der p 23 (ratio 2 = 52%), a finding that would condition the patient's response to AIT.
Diagnosis and final comments	
The patient shows clinical rhinoconjunctivitis and asthma due to house dust mite allergy. Ratio 1 (we‐sIgE/tIgE) is high, supporting an etiopathogenic role for dust mites. Furthermore, in this case, sIgE levels against group 1 and 2 allergens are quite low relative to we‐sIgE levels (ratio 2), suggesting that the patient may be sensitized to other allergens. Consequently, a more comprehensive molecular diagnosis is required. In this case, the patient presented elevated sIgE levels to Der p 23, which is a relevant finding regarding the selection of the specific immunotherapy.	

Abbreviations: c, component; IgE, immunoglobulin E; sIgE, specific IgE; tIgE, total IgE; we, whole extract.

### 3.2 Respiratory allergy (asthma and rhinoconjunctivitis) due to animal dander

A study carried out in patients with cat allergy who received AIT showed that patients with a ratio 1 percentage < 1% were more likely to have lower reactivity on the BAT than those with a ratio 1 > 3%.^31^

To identify the main sensitizer, the ratio 2 (c‐sIgE/we‐sIgE) can be assessed. In the case of dog allergy, if the analysis of ratio to Can f 5/whole dog dander extract reveals that the dog sensitization is mainly due to Can f 5, the patients should be advised to avoid the exposure to male dogs, since this protein is originated from the prostate gland.^32^, ^33^, ^34^ Many patients test positive to pet dander, mainly due to the presence of sIgE to serum albumins and lipocalins (generally associated with more severe forms of the disease).^34^ When selecting the most appropriate AIT, it is important to keep in mind that not all AIT extracts contain all possible allergenic molecules,^35^ so it is clinically relevant to evaluate ratio 2 to determine the primary sensitizing protein.

Table 2 provides a case study to illustrate this clinical scenario.

**TABLE 2:** Case study of patient with respiratory allergy (asthma and rhinoconjunctivitis) to animal dander

Case study	
A 15‐year‐old male patient with persistent asthma and seasonal (spring) rhinoconjunctivitis. The patient has a male dog at home. He presented at the clinic for a suspected dog allergy.	
Skin prick testing	
Positive to extract of dog dander and timothy grass.	
Serological test results	
• tIgE: 112 kU/L	
• we‐sIgE *D. pteronyssinus*: 0.1 kU_A_/L	
• we‐sIgE timothy grass: 8.36 kU_A_/L	
• we‐sIgE dog dander: 35 kU_A_/L	
• sIgE Can f 1: 0.00 kU_A_/L	
• sIgE Can f 2: 0.00 kU_A_/L	
• sIgE Can f 3: 0.00 kU_A_/L	
• sIgE Can f 4: 0.51 kU_A_/L	
• sIgE Can f 5: 4.20 kU_A_/L	
• sIgE Can f 6: 18.5 kU_A_/L	
Ratio analysis	
Ratio 1 we‐sIgE/tIgE	In this case, ratio 1 for dog dander was 31%, indicating sufficient sensitization to support the diagnostic orientation–respiratory allergy –based on the patient's medical history and skin prick testing.
Ratio 2 c‐sIgE/we‐sIgE	Ratio 2 for the component (Can f 5) and whole extract (dog dander) is only 12%; by contrast, ratio 2 for Can f 6 is 53%, indicating that sensitization to dog dander is primarily attributable to this lipocalin.
	The low ratio 2 for Can f 5 (12%) suggested a high likelihood that another molecule was the main sensitizer, a hypothesis that was subsequently confirmed (Can f 6).
Diagnosis and final comments	
The patient presented persistent asthma due to exposure to dog allergens and rhinoconjunctivitis due to sensitization to grass pollens. In this case, ratio 1 (we‐sIgE/tIgE) reveals a clear sensitization to dog dander. Sensitization to Can f 5 suggests that the patient may only be allergic to male dogs (and therefore able to tolerate females). However, the low ratio 2 value for this component suggested that the patient was likely sensitized to other allergenic molecules; in this case, the lipocalin Can f 6. Based on these findings, the patient should be advised to avoid both male and female dogs.	

Abbreviations: c, component; IgE, immunoglobulin E; sIgE, specific IgE; tIgE, total IgE; we, whole extract.

### 3.3 Respiratory allergy (asthma and rhinoconjunctivitis) due to pollens

Although the risk factors for asthma exacerbations are not fully understood,^36^ seasonal variations in asthma‐related hospitalizations are supported by a large body of evidence,^37^ similar to the seasonal patterns commonly observed in allergic rhinoconjunctivitis.^38^ Sensitization to pollens is highly prevalent, affecting 10%–30% of the world population.^39^ For this reason, pollen calendars are an important part of the diagnosis.^40^, ^41^

As with other allergens, to obtain a broader and more accurate picture of the patient's sensitization profile, it is important to determine the tIgE and the we‐sIgE and perform component‐resolved diagnosis (CRD). A study of monosensitized patients (olive or grass pollen) treated with AIT found that ratio 1 (we‐sIgE/tIgE) values > 16.2 were associated with a better response to treatment. Moreover, analysis of the sIgE to tIgE ratio improved the diagnostic sensitivity and specificity when compared to the same assays considered separately.^25^

One study^42^ found that ratio 2 (c‐sIgE/we‐sIgE) was highly useful in orienting the suspected diagnosis towards sensitization to panallergens such as profilin, polcalcin, or cross‐reactive carbohydrate determinants (CCDs) in patients sensitized to any of the major pollen allergens. In that study, subjects with a ratio 2 (Art v 1/mugwort‐sIgE) < 0.5 more often presented IgE to Amb a 1, profilin, polcalcin, and CCDs than subjects with a ratio > 0.5.^42^ This finding suggests that sensitization to other pollens could be secondary to cross‐reactivity and thus not clinically relevant, a finding that could be important in geographic regions with complex pollen exposure, where there is substantial overlap in pollination from several different plant species.

Table 3 provides an example of a case to illustrate this clinical scenario

**TABLE 3:** Case study of patient with respiratory allergy (asthma and rhinoconjunctivitis) to pollen

Case study	
45‐year‐old man living in Córdoba, Spain, who suffers from long‐term seasonal rhinoconjunctivitis and bronchial asthma in spring. He tolerates plant foods.	
Aerobiological study and skin prick tests	
Due to seasonality and the patient's geographical location, the diagnosis is probable sensitization to olive tree pollen and/or grasses. SPT is positive for olive and mugwort pollen, and negative for grasses.	
Serological test results	
• tIgE: 640 kU/L	
• we‐sIgE *Olea europea*: 125 kU_A_/L	
• we‐sIgE *Artemisia vulgaris*: 6 kU_A_/L	
• sIgE Ole e 1: 18.1 kU_A_/L	
• sIgE Ole e 7: 115 kU_A_/L	
• sIgE Ole e 9: 20 kU_A_/L	
Ratio analysis	
Ratio 1 we‐sIgE/tIgE	The patient tested positive for two types of pollens (olive and mugwort). The combination of various factors – geographic area, seasonality, and ratio 1 values – points to olive tree pollen as the symptom trigger. However, it is important to determine the specific sensitizing component allergens. Since olive pollen appears to be clinically relevant (ratio 1 = 19% vs. <1% for mugwort), this finding supports AIT as a part of the overall treatment scheme, with a high probability of obtaining a good response to treatment.
Ratio 2 c‐sIgE/we‐sIgE	If the clinician had only ordered sIgE tests for Ole e 1 (the main allergen in most areas with low pollen exposure) and failed to consider the relationship between the sIgE and we‐sIgE values (ratio 2), the logical conclusion would be that the sensitization is probably attributable to this allergen. If this finding leads the clinician to assume that no further tests are necessary, then he/she may end up overlooking other important sensitizing allergens. In this case, sensitization was mostly due to the lipid transporter protein (LTP) of this pollen, Ole e 7, as evidenced by the high ratio 2 value (92%). Component‐resolved diagnosis provides additional clinical value by raising the possibility that sensitization to mugwort pollen was attributable to cross‐reactivity between the mugwort LTP (Art v 3) and Ole e 7, as has been previously demonstrated between Ole e 7 and peach LTP (Pru p 3).^43^
Diagnosis and final comments	
When prescribing AIT in this case, it is important to bear in mind that Ole e 7 is not quantified in most commercial extracts. Moreover, sensitization to this component is commonly associated with treatment‐related adverse effects.^44^ According to Calderón et al., simultaneous vaccination against two allergens can be clinically effective.^45^ However, in this particular case, it is important to determine whether this is necessary given the differences in sensitization to each pollen.	

Abbreviations: c, component; IgE, immunoglobulin E; LTP, Lipid transfer protein; sIgE, specific IgE; tIgE, total IgE; we, whole extract.

## 4 ALLERGY TO HYMENOPTERA VENOM

In some series, Hymenoptera sting can account for up to 42.8% of all cases of anaphylaxis.^46^ However, the venom's composition is highly heterogeneous due to the low molecular weight of the components responsible for local reactions, while high molecular weight components can provoke systemic reactions.^47^

In this type of allergy, some patients have positive intradermal test and we‐sIgE against *Apis*, *Vespula*, and *Polistes*, even though they have not been stung by all these insects. These findings can be explained by the presence of CCDs, which lead to cross‐reactivity, as well as other components that may also be cross‐reactive.^48^ In these cases, it is possible to distinguish between genuine sensitization and cross‐reactivity by determining ratio 1 values (we‐sIgE/tIgE) against whole extracts of the different species. In practice, high we‐sIgE antibody levels are usually due to genuine sensitization to components but validated cut‐off points are not currently available. A retrospective study of 54 cases of allergy to *Apis mellifera* venom confirmed that ratio 1 for honeybee venom was directly and proportionally correlated with the severity of the reaction. In other words, the ratio was significantly higher in patients with extensive localized reactions vs. those with limited localized reactions and in patients with systemic reactions vs. extensive localized reactions.^49^ Stoevesandt et al. showed that a low sIgE/tIgE ratio might indicate clinically asymptomatic sensitization to Hymenoptera venom or component allergens.^50^

To our knowledge, the diagnostic utility of ratio 2 (c‐sIgE/we‐sIgE) for Hymenoptera venom allergies has not been studied. However, we recommend evaluating this ratio given that the absence of sIgE against allergen components is not a sufficient condition to exclude sensitization to Hymenoptera venom (Table 4). Several studies have evaluated the role of other ratios in the diagnosis of Hymenoptera venom allergy. For example, another serum immunoglobulin – Hymenoptera venom‐specific IgG4 (sIgG4) – may be a relevant marker of exposure to facilitate decision‐making in patients with multiple sensitizations. Levels of sIgG4 have been shown to correlate with specific clinical scenarios. One study found that nonallergic beekeepers present allergen‐specific IgG4 antibody levels to *Apis mellifera* venom that are up to 1000 times higher than the sIgE levels observed in allergic beekeepers; moreover, the number of stings and/or years working as a beekeeper was positively correlated with sIgG4 levels.^51^ Recent studies show that, as a function of AIT treatment duration, the sIgG4/sIgE ratio increases while skin test reactivity decreases, suggesting that this ratio may be a good tool to monitor treatment response.^52^

**TABLE 4:** Case study of patient with Hymenoptera venom allergy

Case study	
45‐year‐old Male. Amateur beekeeper. Anaphylaxis grade IV (Mueller scale) after bee sting	
Intradermal testing	
Positive to whole extract of *Apis mellifera* venom.	
Serological test results	
• tIgE: 112 kU/L	
• we‐sIgE *Apis mellifera*: 45 kU_A_/L	
• sIgE Api m 1: 0.87 kU_A_/L	
• sIgE Api m 2: 0.33 kU_A_/L	
• sIgE Api m 3: 0.01 kU_A_/L	
• sIgE Api m 5: 0.03 kU_A_/L	
• sIgE Api m 10: 52 kU_A_/L	
Ratio analysis	
Ratio 1 we‐sIgE/tIgE	Ratio 1 for *Apis mellifera* is 40.1%, which is sufficient to explain the patient's symptoms after the bee sting.
Ratio 2 c‐sIgE/we‐sIgE	The Api m 1 and Api m 2 ratios are 1.9% and 0.7%, respectively, versus 115% for Api m 10, indicating that this protein is the main sensitizing component. Given that Api m 10 (icarapin) is not quantified in AIT extracts, one option would be to use vaccines known to contain this allergen and/or either increase the immunotherapy maintenance dose or reduce the time span between doses.
Diagnosis and final comments	
The patient has a severe allergy to bee venom due to sensitization to *Apis mellifera* venom. The risk of anaphylaxis – and the potential for therapeutic failure – must be considered when selecting the quantity of this component in the treatment. The tIgE value makes it possible to determine whether the patient is atopic, since reactions after Hymenoptera sting can be found in both atopic and non‐atopic subjects. Furthermore, determination of tIgE can help to better understand the diagnostic value of the various sIgEs. In a hypothetical diagnostic algorithm, tIgE would be measured only after determination of we‐sIgE if the latter value is inconclusive.	

Abbreviations: c, component; IgE, immunoglobulin E; sIgE, specific IgE; tIgE, total IgE; we, whole extract.

A case study is described in Table 4 to illustrate this clinical situation.

## 5 FOOD ALLERGY

Food allergies affect individuals of all ages. The foods responsible for these allergies differ according to each stage and natural history of the pathology.^53^ In Spain, most food‐related allergic reactions are caused by cow milk, eggs, wheat, soy, fruit, nuts, fish, and shellfish.^54^ Genetic predisposition and environmental/geographical factors – which condition dietary habits – play an essential role.^55^, ^56^ The difficulties and risks associated with oral provocation tests^54^, ^55^ underscore the value of in vitro tests.

Ratio 1 (we‐sIgE/tIgE) is useful to assess sensitization to a given food.^5^ There is some evidence to suggest that this ratio could have predictive value. Gupta et al. found that ratio 1 could predict the results of oral food challenges performed to confirm tolerance to peanuts and dried fruits.^57^ By contrast, a retrospective study evaluated 992 oral food challenges in 501 children (mean age, 13 months), finding that ratio 1 did not provide any predictive advantages compared to sIgE levels for the diagnosis of allergies to cow milk, egg, wheat, or soy, leading the authors to conclude that controlled oral food challenges should remain the test of choice.^58^

Ratio 2 (c‐sIgE/we‐sIgE) is useful in foods containing allergens that are underrepresented in the whole extract. For example, Tri a 14 and Tri a 19 are found only in small amounts in whole‐allergen wheat extracts.^3^ In cases with high clinical suspicion despite negative findings on whole extract assays, CRD should be performed as this may reveal reactivity, as demonstrated in a study of Tri a 19.^59^ The utility of ratio 2 for the diagnosis of hazelnut allergy was demonstrated in a study that found that a Cor a 1 to hazelnut‐sIgE ratio >1 was predictive of hazelnut tolerance. This finding has important implications for patients without a clear history of anaphylactic reaction.^60^

Other ratios may also provide valuable data in patients with food allergies. Machinena et al. showed that a tIgE/c‐sIgE ratio ≥3.75 for cow milk protein was a reliable predictor of tolerance, thus potentially eliminating the need for avoidance diets.^61^ A paediatric population study found that the ovalbumin‐sIgE/tIgE ratio had a significantly higher area under the curve than the observed for sIgE or SPT, indicating a better predictive capacity for raw egg tolerance.^62^ In a study conducted to assess α‐Gal‐mediated red meat allergies, 131 individuals with a suspected allergy were compared to 26 controls. The results showed that sIgE values > 5.5 kU_A_/L and a c‐sIgE/tIgE ratio >2.12% were predictive of meat allergy, with a 95% probability.^63^ In peanut allergies, a study found that elevated IgG and IgG4 to tIgE ratios were predictive of less severe reactions, but sIgE for Ara h 2 was a better predictor of peanut allergy.^64^ Another study in a paediatric population (*n* = 207) reached similar conclusions, finding that serum specific Cor a 14 and Ara h 2 IgE levels were better predictors of allergy severity (in this case, to hazelnuts and peanuts) than component‐specific to tIgE ratios.^65^

One study found that the c‐sIgE/sIgG4 ratio for egg components could predict tolerance in allergic patients,^66^ similar to the findings of another study showing that the same ratio (c‐sIgE/sIgG4) for casein and β‐lactoglobulin was predictive of tolerance to cow milk.^67^ In line with these results, another study found that ovalbumin‐specific sIgG4 was an independent predictor of tolerance to fresh egg.^68^

Table 5 describes a case study to illustrate this clinical scenario.

**TABLE 5:** Case study of patient with a food allergy

Case study	
A 26‐year‐old woman with a family and personal history of egg allergy and seasonal allergic rhinitis to birch pollen. The patient consulted for generalized urticaria, facial angioedema, and gastrointestinal symptoms (without cardiovascular compromise), after eating pasta, beef, and hazelnut ice cream	
Skin prick testing	
Positive for hazelnut extract and birch pollen. Negative for beef and wheat extract	
Serological test results	
• tIgE: 42 kU/L	
• we‐sIgE hazelnut: 0.27 kU_A_/L	
• sIgE Cor a 1: 0.30 kU_A_/L	
• sIgE Cor a 8: 0.0 kU_A_/L	
• sIgE Cor a 9: 0.0 kU_A_/L	
• sIgE Cor a 14: 0.0 kU_A_/L	
• we‐sIgE beef: 0.04 kU_A_/L	
• sIgE α‐Gal (galactose‐α‐1,3‐galactose): 2.3 kU_A_/L	
• sIgE Bos d 6 bovine serum albumin: 0.0 kU_A_/L	
• we‐sIgE wheat: 0.0 kU_A_/L	
• sIgE Tri a 14: 0.0 kU_A_/L	
• sIgE Tri a 19: 0.0 kU_A_/L	
Ratio analysis	
Ratio 1 we‐sIgE/tIgE	Although the patient was sensitized (sIgE) to hazelnut extract, with a relatively low tIgE, the we‐sIgE/tIgE ratio was only 0.7%. Based on previous reports,^45^ this finding is not suggestive of clinical relevance.
Ratio 2 c‐sIgE/we‐sIgE	Further tests were performed to assess sensitization to other allergenic components of hazelnut. These tests suggested that sensitization to hazelnut extract was likely attributable to Cor a 1, PR‐10/Bet v 1 homologue of hazelnut, since the sIgE value for this component was virtually the same as for the whole hazelnut extract. According to Lange et al.,^60^ ratio 2 values so close to 1 (rCor a 1/hazelnut extract sIgE) is not suggestive of clinical reactivity, especially if the patient's sensitization to birch pollen (Bet v 1) is also considered, as this suggests that sensitization to Cor a 1 may be due to cross‐reactivity.
	In this case, the patient's sIgE sensitization to α‐Gal was 2.3 KUA/L, with a ratio of 5750% (we‐sIgE/tIgE). According to Mabelane et al.,^63^ both results are highly suggestive of clinical relevance.
Diagnosis and final comments	
In a patient sensitized to α‐Gal, this finding could explain anaphylaxis after consuming beef. The weak sensitization to hazelnut PR‐10 could be interpreted in the context of sensitization to birch pollen (Bet v 1/PR‐10). Confirmatory diagnosis should be made by an oral challenge test, which remains the gold standard.	

Abbreviations: c, component; IgE, immunoglobulin E; sIgE, specific IgE; tIgE, total IgE; we, whole extract.

## 6 ANAPHYLAXIS

The most recent EAACI guidelines^69^ define anaphylaxis as a potentially life‐threatening condition but, due to practical and ethical challenges, there is a paucity of robust evidence about how to diagnose and manage this. The annual incidence of anaphylaxis ranges from 50 to 112 episodes per 100,000 people; the incidence in children under age 4 is three times higher than in the general population.^70^ From 1992 to 2012 (20 years), anaphylaxis‐related hospitalizations increased six‐fold.^71^ According to a study published in 2014, the leading causes of anaphylaxis in adults were drugs (34% of cases), foods (31%), Hymenoptera venom (20%), aeroallergens (7.5%), latex (2.6%), and ‘other’ causes (12.2%).^72^

Diagnosis of food‐related anaphylaxis or Hymenoptera venom allergy should follow the same procedures described in the previous sections. In addition, baseline and acute serum tryptase levels should be determined since patients with severe anaphylaxis (grade IV) have significantly higher levels of tryptase than those with mild to moderate anaphylaxis.^73^ Furthermore, an increase in serum tryptase values > 20% above baseline levels +2 μg/L serves as a biomarker for MC activation and is one of the criteria applied for MCAS diagnosis.^74^, ^75^

A study assessed 171 patients with a history of immediate reactions to β‐lactams compared to 122 healthy controls, finding that a β‐lactam‐sIgE [hapten c1 (penicilloyl G), c2 (penicilloyl V), c5 (ampicilloyl), c6 (amoxicilloyl)] to tIgE ratio ≥ 0.002 had a positive predictive value of 92.5%, thus enabling the identification of genuinely reactive patients, even among individuals with high tIgE levels (>200 kU/L).^76^

As mentioned above (food allergy section), ratio 2 can be useful in patients with anaphylactic reactions due to poorly represented components in the total extracts, such as Tri a 14 and Pru p 7.^3^

A case study is provided in Table 6 to better illustrate this clinical scenario.

**TABLE 6:** Case study of patient with anaphylaxis

Case study	
19‐year‐old woman consulting after anaphylactic reaction while exercising after eating a tangerine. The patient had a history of urticaria episodes after eating a peach and anaphylaxis after eating grapes and drinking alcohol.	
Skin prick testing	
Positive for whole extract of peach skin	
Serological test results	
• tIgE: 341 kU/L	
• we‐sIgE peach: 1.66 kU_A_/L	
• we‐sIgE orange: 0.72 kU_A_/L	
• sIgE Pru p 3: 0.00 kU_A_/L	
• sIgE Pru p 7: 1.30 kU_A_/L	
Ratio analysis	
Ratio 1 we‐sIgE/tIgE	In this case, no commercial assays are available to test for tangerine reactivity, so an orange was used instead as a citrus substitute.
Ratio 2 c‐sIgE/we‐sIgE	Sensitization to peach extract is mainly attributable to Pru p 7, which accounts for approximately 78% of the we‐sIgE.
Diagnosis and final comments	
Patient sensitized to peach peamaclein (Pru p 7), an allergen from the family of proteins regulated by gibberellin with cross‐reactivity with orange (Cit s 7). In this case, we suspected sensitization to a peach allergen other than LTP due to the discrepancy between the sIgE to Pru p 3 and the we‐sIgE. This finding again underscores the importance of interpreting the c‐sIgE to we‐sIgE (ratio 2). Sensitization to profilins or homologues of Bet v 1 was ruled out by ImmunoCAP ISAC assay (Thermo Fisher Scientific, Sweden).	

Abbreviations: c, component; IgE, immunoglobulin E; sIgE, specific IgE; tIgE, total IgE; we, whole extract.

## 7 DISCUSSION

Molecular diagnostics represents a major advance in the management of allergic patients. In routine clinical practice, the optimal use of these diagnostic tools may not always be clear due to the lack of validated protocols, insufficient clinical experience with these tools, and because the test results frequently need to be interpreted indirectly. However, most professionals involved in allergy diagnosis are highly motivated and interested in learning how to apply these techniques to improve the diagnosis and treatment of their patients.

Like all techniques, molecular diagnosis has limitations. One significant limitation is that even though numerous different allergens can provoke an allergic reaction, only a limited number can be tested. Another limitation is the diagnostic complexity associated with the molecular diagnosis. While the capacity to detect specific allergenic components is probably the most notable innovation in this field in recent years, and a major improvement in diagnostic efficiency, it has also greatly increased the complexity of the diagnostic process. Moreover, the presence of a given allergenic molecule does not always explain the patient’s whole sensitization profile; in fact, in many cases, the sensitization profile and clinical expression of the allergy are not correlated.^77^ Consequently, diagnostic resources are sometimes utilized, but without taking advantage of their full potential.

In the context described above, it is clear that novel evidence‐based tools are needed to overcome the limitations associated with molecular diagnosis. For all these reasons, we believe that ratio analysis is an essential tool to identify the allergenic components that indeed underlie the clinical manifestations of an allergic reaction. This implies the need to use a two‐pronged approach to diagnosis (i.e., complete medical history and comprehensive in vitro diagnostic testing). This approach would significantly improve diagnostic accuracy and positively impact QoL. Despite the emergence and continuous growth of molecular diagnostics, it is still important to assess we‐sIgE reactivity because molecular findings lose value if not analysed in the context of sensitization to the whole extract and tIgE antibody levels.

**FIGURE 1:**
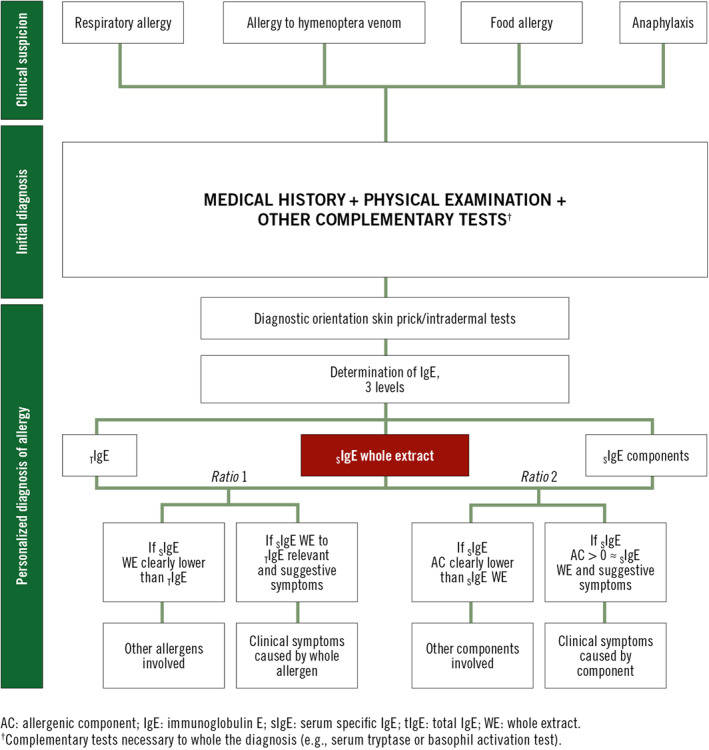
Implementation of ratio analysis in a personalized allergy diagnostic algorithm. Once a clinical suspicion of allergy exists, the initial diagnosis begins with a medical history, physical examination, and other complementary tests. The posterior use of the ratio analysis allows for a personalized diagnosis of allergy

Expert‐based consensus statements, such as the present document, can be particularly useful when the existing evidence base is limited. Such documents fill a critical knowledge gap, thus helping to improve health care services.^78^ To facilitate the clinical application of the ratios described here, we have developed a series of recommendations (Table 7) and a diagnostic algorithm (Figure 1) based on the literature review and case studies presented in this document, with the ultimate aim of improving allergy management in routine clinical practice. However, further prospective observational studies with a large number of patients would strengthen the clinical validation of the ratio’s application described here.

**TABLE 7:** Recommendations for the clinical application of ratios in allergy diagnosis

Recommendation	LA
General recommendations for ratio 1: Determine whole extract serum‐sIgE and tIgE levels and then calculate the relationship between these two values (ratio 1: we‐sIgE/tIgE) before clinical decision‐making. This ratio may be particularly useful in assessing patients with low tIgE levels.	82.14%
A positive result for sIgE to a whole allergen extract (e.g., food or inhalants) should be interpreted in the context of tIgE levels before making any clinical decisions.	82.14%
General recommendations for ratio 2: First, calculate ratio 2 (component sIgE [c‐sIgE]/whole extract sIgE [we‐sIgE]). This ratio can be used to determine the involvement of a given allergenic component, especially minor allergens.	75.00%
Positive results based on low we‐sIgE values (or values below the cut‐off point in most diagnostic assays) should be complemented with molecular diagnosis and assessment of ratio 2 in cases with high clinical suspicion.	78.57%
If considering allergen‐specific immunotherapy (AIT) for ≥ one inhalant allergens (at least pollens), we‐sIgE testing should be complemented with available molecular tests for component allergens. Ratio 2 should be evaluated to identify the primary sensitizer to determine if the patient is a candidate for AIT.	75.00%
It is recommended to include all relevant diagnostic components. The ratio 2 can be useful to determine whether AIT with available extracts is indicated.	82.14%
Other recommendations	
Determination of sIgE against the whole allergen extract is recommended since the lack of sIgE against component allergens is not sufficient to rule out the diagnosis given that not all components have been described and/or because currently available assays do not include those components.	100.00%
In cases involving allergies to Hymenoptera venom or certain foods, component testing should be performed even if the we‐sIgE assay is negative since a negative result is not sufficient to rule out an allergy diagnosis (the extract may not contain the sensitizing allergen).	92.85%

Abbreviations: AIT, Allergen‐specific immunotherapy; c, component; IgE, immunoglobulin E; LA, Level of agreement; sIgE, specific IgE; tIgE, total IgE; we, whole extract (see Supporting information).

It is important to emphasise that no defined cut‐off points have been established for these ratios; that is, there are no clear numerical values to unequivocally indicate that a given whole‐allergen extract, or its components are the main cause of the allergic reaction. Although cut‐off thresholds are not always necessary because the evaluation of the individual test results (sIgE and tIgE) and the relationship between these values (ratios), together with the patient’s clinical history provides important information, future studies to establish cut‐off values would improve diagnostic accuracy.^79^

Ratio analysis offers the potential to improve both the diagnosis and management of allergic patients. The clinical applicability of ratios has been supported by the 28 allergy international experts who participated in the Delphi method. With this work we would like to provide allergists with a series of validated recommendations to encourage them to use ratios as a complementary diagnostic tool.

Although much progress has been made in recent years, more studies are needed to understand better how to interpret the relationship between sIgE and sIgG4 levels to manage allergic diseases. This is the only way to fully exploit these measurements to determine if they can be used as biomarkers to facilitate allergy diagnosis and treatment.

## CONFLICT OF INTEREST

The authors have received consulting fees and financial support from Thermo Fisher Scientific to attend workshops related to the preparation of this article.

## AUTHOR CONTRIBUTION

Olga Luengo Sánchez: Conceptualization; Equal, Data curation; Equal, Formal analysis; Equal, Investigation; Equal, Methodology; Equal, Writing‐original draft; Equal, Writing‐review & editing; Equal, Mariona Pascal: Conceptualization; Equal, Data curation; Equal, Formal analysis; Equal, Investigation; Equal, Methodology; Equal, Writing‐original draft; Equal, Writing‐review & editing; Equal, C. Moreno: Conceptualization; Equal, Data curation; Equal, Formal analysis; Equal, Investigation; Equal, Methodology; Equal, Writing‐original draft; Equal, Writing‐review & editing; Equal, Ignacio Davila: Conceptualization; Equal, Data curation; Equal, Formal analysis; Equal, Investigation; Equal, Methodology; Equal, Writing‐original draft; Equal, Writing‐review & editing; Equal, Ana Isabel Tabar: Conceptualization; Equal, Data curation; Equal, Formal analysis; Equal, Investigation; Equal, Methodology; Equal, Writing‐original draft; Equal, Writing‐review & editing; Equal, Joan Bartra: Conceptualization; Equal, Data curation; Equal, Formal analysis; Equal, Investigation; Equal, Methodology; Equal, Writing‐original draft; Equal, Writing‐review & editing; Equal, Moisés Labrador Horrillo: Conceptualization; Equal, Data curation; Equal, Formal analysis; Equal, Investigation; Equal, Methodology; Equal, Writing‐original draft; Equal, Writing‐review & editing; Equal.

## Supporting information

Supporting Information S1
