# Supplementary material for: Integration of in vitro allergy test results and ratio analysis for the diagnosis and treatment of allergic patients (INTEGRA)
Source: Clin Transl Allergy. 2021 Sep 16;11(7):e12052. doi: 10.1002/clt2.12052 (PMC9082998; doi:10.1002/clt2.12052)
Supplement: Supplementary file 1 — Supporting Information S1 [file CLT2-11-e12052-s001.docx]

**SUPPORTING INFORMATION**

The authors of the manuscript selected the members of the Delphi panel, who collaborated as external advisors validating and/or suggesting modifications to the recommendations. The selection criteria were based taking into consideration their experience in the allergy and molecular diagnosis field, and also considering the field of work, years of experience, and authorship of peer-reviewed manuscripts.

The final external advisor panel included 26 allergists, 1 internal medicine physician, and 1 pediatric allergist. The years of clinical practice ranged from 7 to 31 years.

A detailed view of the scores and percentages obtained in the Delphi questionnaire is listed below. All the recommendations were rated by the total number of panellists.

**Section 1. RECOMMENDATIONS 1^st^ ROUND**

*Recommendation 1.* Determine whole extract serum-sIgE and tIgE levels and then calculate the relationship between these two values (ratio 1: we-sIgE/tIgE) before clinical decision-making. This ratio may be particularly useful in assessing patients with low tIgE levels.

| **Score** | **1** | **2** | **3** | **4** |
| --- | --- | --- | --- | --- |
| **Total of answers per score** | 1 | 4 | 9 | 14 |
| **Percentage (%)** | 3.57 | 14.29 | 32.14 | 50 |
| **Total percentage disagreement/agreement (%)** | **17.86** | | **82.14** | |

*Recommendation 2.* A positive result for sIgE to a whole allergen extract (e.g., food or inhalants) should be interpreted in the context of tIgE levels before making any clinical decisions

| **Score** | **1** | **2** | **3** | **4** |
| --- | --- | --- | --- | --- |
| **Total of answers per score** | 1 | 4 | 10 | 13 |
| **Percentage (%)** | 3.57 | 14.29 | 35.71 | 46.43 |
| **Total percentage disagreement/agreement (%)** | **17.86** | | **82.14** | |

*Recommendation 3.* First, calculate ratio 2 (component sIgE [c-sIgE]/whole extract sIgE [we-sIgE]). This ratio can be used to determine the involvement of a given allergenic component, especially minor allergens

| **Score** | **1** | **2** | **3** | **4** |
| --- | --- | --- | --- | --- |
| **Total of answers per score** | 2 | 5 | 8 | 13 |
| **Percentage (%)** | 7.14 | 17.86 | 28.57 | 46.43 |
| **Total percentage disagreement/agreement (%)** | **25** | | **75** | |

*Recommendation 4.* Positive results based on low we-sIgE values (or values below the cut-off point in most diagnostic assays) should be complemented with molecular diagnosis and assessment of ratio 2 in cases with high clinical suspicion

| **Score** | **1** | **2** | **3** | **4** |
| --- | --- | --- | --- | --- |
| **Total of answers per score** | 0 | 6 | 8 | 14 |
| **Percentage (%)** | 0 | 21.43 | 28.57 | 50 |
| **Total percentage disagreement/agreement (%)** | **21.43** | | **78.57** | |

*Recommendation 5.* If considering allergen-specific immunotherapy (AIT) for ≥ one inhalant allergens (at least pollens), we-sIgE testing should be complemented with available molecular tests for component allergens. Ratio 2 should be evaluated to identify the primary sensitizer to determine if the patient is a candidate for AIT.

| **Score** | **1** | **2** | **3** | **4** |
| --- | --- | --- | --- | --- |
| **Total of answers per score** | 3 | 4 | 5 | 16 |
| **Percentage (%)** | 10.71 | 14.29 | 17.86 | 57.14 |
| **Total percentage disagreement/agreement (%)** | **25** | | **75** | |

*Recommendation 6*. It is important to include the widest possible diagnostic component panel and to assess ratio 2 to determine whether immunotherapy—a critical treatment for quality of life—is indicated

| **Score** | **1** | **2** | **3** | **4** |
| --- | --- | --- | --- | --- |
| **Total of answers per score** | 3 | 7 | 7 | 11 |
| **Percentage (%)** | 10.71 | 25 | 25 | 39.29 |
| **Total percentage disagreement/agreement (%)** | **35.71** | | **64.29** | |

*Recommendation 7.* Determination of sIgE against the whole allergen extract is recommended since the lack of sIgE against component allergens is not sufficient to rule out the diagnosis given that not all components have been described and/or because currently available assays do not include those components

| **Score** | **1** | **2** | **3** | **4** |
| --- | --- | --- | --- | --- |
| **Total of answers per score** | 0 | 0 | 6 | 22 |
| **Percentage (%)** | 0 | 0 | 21.43 | 78.57 |
| **Total percentage disagreement/agreement (%)** | **0** | | **100** | |

*Recommendation 8.* In cases involving allergies to Hymenoptera venom or certain foods, component testing should be performed even if the we-sIgE assay is negative since a negative result is not sufficient to rule out an allergy diagnosis (the extract may not contain the sensitizing allergen).

| **Score** | **1** | **2** | **3** | **4** |
| --- | --- | --- | --- | --- |
| **Total of answers per score** | 0 | 2 | 9 | 17 |
| **Percentage (%)** | 0 | 7.14 | 32.14 | 60.71 |
| **Total percentage disagreement/agreement (%)** | **7.14** | | **92.86** | |

**Section 2. RECOMMENDATION 2nd ROUND**

*Recommendation 6.* It is recommended to include all relevant diagnostic components. The ratio 2 can be useful to determine whether AIT with available extracts is indicated.

| **Score** | **1** | **2** | **3** | **4** |
| --- | --- | --- | --- | --- |
| **Total of answers per score** | 2 | 3 | 17 | 6 |
| **Percentage (%)** | 7.14 | 10.71 | 60.71 | 21.43 |
| **Total percentage disagreement/agreement (%)** | **17.86** | | **82.14** | |
